# Non-coding RNAs shuttled via exosomes reshape the hypoxic tumor microenvironment

**DOI:** 10.1186/s13045-020-00893-3

**Published:** 2020-06-05

**Authors:** Wenyu Wang, Youngjin Han, Hyun A Jo, Juwon Lee, Yong Sang Song

**Affiliations:** 1 Interdisciplinary Program in Cancer Biology, Seoul National University College of Medicine, Seoul, 03080 Republic of Korea; 2 Cancer Research Institute, Seoul National University College of Medicine, Seoul, 03080 Republic of Korea; 3 Biomodulation, Department of Agricultural Biotechnology, Seoul National University, Seoul, 03080 Republic of Korea; 4 Department of Obstetrics and Gynecology, Seoul National University College of Medicine, Seoul, 03080 Republic of Korea

**Keywords:** Exosomes, Hypoxia, Tumor microenvironment, MiRNA, LncRNA, Non-coding RNA

## Abstract

Exosomes are small extracellular vesicles secreted by almost all the cells. Molecular cargos of exosomes can partially reflect the characteristics of originating cells. Exosome-mediated cell-to-cell interactions in the microenvironment are critical in cancer progression. Hypoxia, a key pro-cancerous feature of the tumor microenvironment, alters the releasing and contents of exosomes. A growing body of evidence shows that hypoxia induces more aggressive phenotypes in cancer. Of note, non-coding RNAs shuttled in hypoxic tumor-derived exosomes have been demonstrated as fundamental molecules in regulating cancer biology and remodeling tumor microenvironment. Furthermore, these hypoxic tumor-derived exosomal non-coding RNAs can be detected in the body fluids, serving as promising diagnostic and prognostic biomarkers. The current review discusses changes in cancer behaviors regulated by exosomes-secreted non-coding RNAs under hypoxic conditions.

## Background

Cancer cells persistently interact with other cell types in the tumor microenvironment. Cells cohabiting in a tumor niche are affected significantly by surrounding factors [1]. Low oxygen status, termed as hypoxia, is one of the most important characteristics of solid tumors. Hypoxia can elicit fundamental changes in cancer cells and affect cell-to-cell communications. Autocrine and paracrine signaling through cytokines and chemokines have been studied intensively in molecular oncology for the last decades [2, 3]. Emerging studies suggest exosomes secreted by different cell types are actively involved in modulating cancer cell phenotypes and dictating cancer hallmarks.

Exosomes contain cytosolic cargos of donor cells, assisting the membrane-protected cargos to travel a long distance. Among many other cytosolic cargos found in exosomes, non-coding RNAs (ncRNAs) including microRNAs (miRNAs), long non-coding RNAs (lncRNAs), and circular RNAs (circRNAs) are most intensively studied in that they act as vital regulators in transcriptional and post-transcriptional levels. Investigating the effects of exosomal non-coding RNAs in the hypoxic tumor microenvironment is critical to elucidate the key underlying mechanisms of cancer progression.

Collectively, this review highlights recent findings of exosome-facilitated cancer progression in the hypoxic tumor microenvironment with a particular focus on non-coding RNAs and proposes clinical applications of exosomal non-coding RNAs as biomarkers.

### **Tumor microenvironment and hypoxia**

Accumulative evidence suggests that tumor cells behave differently depending on extrinsic factors of the surrounding microenvironment. Distinct types of cells including immune cells, fibroblasts, and endothelial cells interactively communicate to facilitate cancer progression [4]. Additionally, pH, reactive oxygen species (ROS), inflammation, components in the extracellular matrix (ECM), and hypoxia can influence the metabolism and aggressiveness of cancer [5–8]. Therefore, the various interplay between cellular and non-cellular factors in the tumor microenvironment may serve as potential therapeutic targets in the clinical settings.

Irregular vessel formation and the rapid proliferation of cancer cells create hypoxic conditions in malignant tumors. In this regard, hypoxia is a common feature in the microenvironment of most solid tumors [9]. Clinical studies have revealed that overexpression of hypoxia-induced genes is associated with poor prognosis in many cancer types including pancreatic, lung, breast, prostate, and ovarian cancer [10–14]. Besides, plentiful in vitro and in vivo experimental data have suggested that hypoxia orchestrates malignant phenotypes of cancer cells through activation of multiple oncogenic signaling pathways. Transcription factors and epigenetic regulators can concertedly exert reinforcement of oncogenic signaling pathways, controlling the expression of numerous genes under hypoxia. Nevertheless, interactions between cancerous cells and non-cancerous cells could be further invigorated in the hypoxic tumor microenvironment. Cancer cells stimulated by hypoxia manifest increased drug-resistance, tumorigenesis, angiogenesis, invasiveness, and immune suppression [15].

Several decades ago, oxygen sensing mechanisms at the molecular level had been discovered that some transcription factors play a central role in tissues in response to low oxygen tension (< 10 mmHg) [16]. The vital proteins involved in the process of cellular adaptation under hypoxia are hypoxia-inducible factor-1 (HIF-1), prolyl hydroxylase (PHD), and von Hippel-Lindau (VHL). HIF-1α is a transcription factor constitutively activated in response to hypoxia [17]. Under normal oxygen tension (45–65 mmHg) at peripheral tissues, PHD is activated, adding a hydroxyl group to HIF-1α at a proline residue. Hydroxylated HIF-1α is then subjected to degradation through ubiquitination, mediated by the VHL complex. Unlike HIF-1α, its binding partner, HIF-1β is stably expressed even at the high oxygen tension. Under hypoxic conditions, the accumulated dimer of HIF-1α and HIF-1β bind to hypoxic response elements (HREs) of various genes in the nucleus. Activated HREs are closely associated with oncogenic phenotypes such as proliferation, invasion, epithelial-mesenchymal transition (EMT), and metabolic reprogramming [16]. Furthermore, recent studies have illustrated that myriad epigenetic modifications are involved in hypoxic signals through histone modifications and DNA methylation [18, 19].

The hypoxic conditions also affect interactions between cancerous and non-cancerous cells in the tumor microenvironment. Hypoxia-induced overexpression of programmed death-ligand 1 (PD-L1) in cancer cells disabled cytotoxic functions of programmed cell death protein 1(PD-1) positive activated T lymphocytes [20, 21]. Moreover, HIF-1α promoted the overexpression of PD-L1 in myeloid-derived suppressor cells (MDSCs) and macrophages, neutralizing anti-cancer immunity in the tumor microenvironment [22]. Additionally, hypoxia upregulated V-domain Ig suppressor of T cell activation (VISTA) in MDSCs, thereby suppressing T cell activity [23]. CD47 enriched at the plasma membrane of hypoxic tumors inhibited the phagocytic activity of macrophages [23, 24]. Along with hypoxia-induced changes in the tumor immune microenvironment, hypoxia can assist tumor growth by reprogramming fibroblasts. In the study which utilized the cancer-associated fibroblast (CAF)-endothelial cell co-culture model, hypoxic CAFs promoted angiogenesis through NCBP2-AS2-mediated vascular endothelial growth factor A (VEGFA) secretion [25]. In line with the result from the CAF-endothelial co-culture study, another study demonstrated that CAF induced angiogenesis via recruitment of HIF-1α and G-protein estrogen receptor (GPER) to the promoter region of VEGF [26]. Moreover, pancreatic CAFs produced more insulin-like growth factor 1 (IGF1), and cancer cells increased the expression of IGF1 receptor (IGF1R) in response to hypoxia. This specific hypoxia-potentiated CAF-cancer cell communication promoted the metastatic ability of pancreatic cancer cells [27]. A multi-center study from Norwegian hospitals suggested that upregulation of miRNA-210, a hypoxia-induced miRNA, in CAFs but not in cancer cells was negatively correlated with the prognosis of prostate cancer patients [28]. Therefore, hypoxic responses of both cancerous and non-cancerous cells are vital determining factors of cancer progression.

Transcriptomic and epigenetic landscapes of a tumor are vastly changed by hypoxia. As recent studies have underscored the importance of exosomes as a critical mode of cell-to-cell communication, it would be worthwhile to unveil how exosomal non-coding RNA signaling regulates the hypoxic tumor microenvironment.

### **Exosomes and non-coding RNAs**

Extracellular vesicles (EVs) are secreted by almost all cell types into the extracellular space. EVs are classified into microvesicles, apoptotic bodies, exosomes, etc., according to their intracellular origin, size, and biogenesis [29]. Exosomes are endogenous vehicles with a size of 40–150 nm in diameter. Endocytic vesicles produced in the plasma membrane can form early-endosomes by continuous endocytosis and develop into late-endosomes. Late-endosomes bud inwards to form multi-vesicular bodies (MVBs), and the fused MVBs with the plasma membrane enable the release of intraluminal vesicles (ILVs) into extracellular space, called exosomes [30]. The endosomal-sorting complex required for transport (ESCRT) machinery is necessary for exosome formation at endosomes. It is complicated protein machinery comprised of four protein ESCRTs (0 through III) regulating MVB formation, vesicle budding, and cargo sorting [31]. However, some studies have revealed an ESCRT-independent exosomal cargo sorting manner, suggesting that the mechanisms are broader and more intricate.

Exosomes are mediators of cell-cell interactions in that they are capable of delivering functional mRNAs, microRNAs (miRNAs), DNAs, and proteins to recipient cells altering their physiological and pathological functions. These exosomal cargos are enclosed inside the double membrane and are stable to environmental factors such as nucleases, proteases, and oxidative stress so that they can be delivered to recipient cells in an efficient and intact manner [32, 33]. Cancer cell-derived exosomes affect cancer progression such as proliferation, drug resistance, and metastasis [34]. Meanwhile, these tumor-derived exosomes also have a significant impact on various stromal cells in the tumor microenvironment. They are involved in the function of endothelial cells, the polarization of macrophages, regulation of T cells, and suppression of natural killer cells (NK cells) activity and other biological activities [35–38]. Stromal cell-secreted exosomes, in turn, can support the malignant phenotypes of cancer cells. For instance, tumor-associated macrophage (TAM)-derived exosomes containing functional factors promoted migration and invasion in breast cancer [39]. CAF-derived exosomes conferred chemoresistance to ovarian cancer cells [40]. Therefore, exosomes may play a pivotal role in the inter-tumor cell, inter-stromal cell, and tumor-stromal cell interactions.

Additionally, exosomes have been detected in almost all types of body fluids such as blood, urine, saliva, breast milk, and ascites. They can be used as biomarkers to diagnose diseases in non-invasive or minimally invasive ways [41]. Furthermore, exosomes can be used as natural nano-carriers for therapeutic applications that can efficiently deliver various signaling molecules. Exosomes show similar structures to a bilayer of lipids of cell membranes because they fuse to the plasma membrane during secretion. Recent researches have shown the possibility that exosome-mediated delivery of small interfering RNAs (siRNAs), antioxidants, anticancer drugs, and CRISPR/Cas9 system via low immunogenicity [42–45].

RNAs are classified into protein-coding RNAs and non-coding RNAs according to their protein-coding abilities. Based on the Francis Crick’s “the central dogma of molecular biology,” many studies have focused on processes of protein production through messenger RNA (mRNA) and constituent RNAs such as ribosomal RNAs (rRNAs) or translators of codon sequence (tRNA) [46, 47]. However, results from human genome sequencing found that only ~ 200,000 RNAs could encode proteins, comprising only 2% of the genomes [48]. In 1965, scientists discovered a new type of regulatory non-coding RNAs that do not function through protein translation [49]. Non-coding RNAs are divided based on the number of nucleotides constituting the RNAs: small non-coding RNAs including miRNAs, siRNAs, piwi-interacting RNAs (piRNAs) composed of less than 200 nucleotides, and lncRNAs with more than 200 nucleotides in size [50]. In addition, another type of non-coding RNAs highly represented in the eukaryotic transcriptome is circRNAs, which form covalently closed continuous loop structures, unlike the abovementioned linear RNAs. CircRNAs are relatively stable compared to linear non-coding RNAs. Exosomes contain a variety of RNA species, among which miRNAs are the most abundant and surely most intensively studied while lncRNAs and circRNAs are also becoming research hotspots now. Some qualitative and quantitative assays have revealed the asymmetric distribution of RNAs between cells and cell-derived exosomes. This phenomenon has boosted many interesting hypotheses, suggesting that RNA molecules are not randomly packaged in exosomes but with a set of sorting systems involved.

A single mammalian cell carries approximately 100,000 endogenous miRNAs, while a single exosome contains about 500 miRNAs. Pigati et al. found that the abundance of about 66% of extracellular miRNAs well reflected the corresponding abundance of miRNAs in the cells [51]. van Balkom et al. also reported that the most abundant exosomal miRNAs closely corresponded with the most abundant cellular miRNAs [52]. These findings suggest that the majority of high expressed miRNAs are encapsulated and secreted via exosomes passively for an osmotic-like effect. However, there was still a category of miRNAs that might be selectively retained or released. For example, more than 90% of the mature miR-451, which is functioned as a tumor suppressive gene in breast cancer, was found to be exported into extracellular space [52]. In addition, Hannafon et al. observed that miR-451, miR-122, miR-1246, and miR-21 were more enriched in the breast cancer cell-derived exosomes than normal epithelial cell-derived exosomes. Intriguingly, oncogenic miR-1246 and miR-21 showed consistent high abundancy in the cells while tumor suppressive miR-451 and miR-122 were downregulated in cellular expression [53]. Based on these studies, it can be inferred that apart from the passive osmotic-like pattern, the function-dependent selective mechanism may be present when RNAs are secreted via exosomes. Cells might tend to secrete RNAs that are unnecessary, advantageous, or even harmful for sustaining cell properties. Ragusa et al. further strengthened and developed this point. They found that in addition to tumor suppressive miRNAs, immunosuppressive miRNAs were also highly abundant in exosomes compared to cells, which indicated that RNAs might be selectively released into the tumor microenvironment to influence the immune response [54].

Some lipids and proteins have been demonstrated to be involved in sorting specific non-coding RNAs into exosomes. The neural sphingomyelinase 2 (nSMase2) was reported to be associated with the secretion of exosomes, which was also the first molecule found to guide miRNAs into exosomes. The expression of nSMase2 was positively related to the level of exosomal miRNAs [55]. Besides, some important proteins might also impact the RNA selective sorting process. Argonaute 2 (Ago2), a component of the miRNA-induced silencing complex (miRISC), was implicated in the assortment of several miRNAs into exosomes. Additionally, Alix, an accessory protein of ESCRT, was reported to be involved in the release of exosomal miRNAs from liver stem-like cells through interacting with Ago 2 [56]. It has been reported that hypoxia could inhibit the Ago2 degradation by mediating hydroxylation of Ago2 by C-P4H(I) and increase the various functions of it, indicating the possible regulative role of hypoxia in sorting miRNAs into exosomes [57]. Villarroya-Beltri et al. identified that some short motifs were overexpressed in exosomal miRNAs and mRNAs. The protein heterogeneous nuclear ribonucleoprotein A2B1 (hnRNPA2B1) could bind to those motifs and modulate RNAs loading into exosomes. Moreover, this process was controlled by the sumoylation of hnRNPA2B1 [58]. Besides, hnRNPA2B1 was also reported to be involved in the encapsulation of lncARSR into exosomes [59]. Kossinova et al. detected several structural motifs enriched in exosomal lncRNAs and mRNAs using bioinformatics approaches. Cytosolic Cold shock protein YB-1 and RNA methyltransferase NSUN2 could recognize these motifs and mediate sorting specific lncRNAs and mRNAs into exosomes [60]. YB-1 was reported to be physically interacted with HIF-1α and regulate the transcription of hypoxia-dependent genes [61]. In addition, YB-1 could enhance the expression of HIF-1α and promote the invasion and metastasis in sarcoma [62]. Li et al. showed more abundant circRNAs were present in exosomes compared to the source cells by RNA-sequencing analyses for the first time. The level of exosomal circRNAs and cellular circRNAs were only moderately correlated, suggesting that certain circRNAs might be actively incorporated into exosomes while some might be selectively retained in cells. CircRNAs have been reported to be capable of binding to miRNAs working as miRNA sponges. The authors also demonstrated that the selective sorting of circRNAs was associated with the relevant miRNAs [63]. Overall, non-coding RNAs with certain sequences may favor themselves loading into exosomes, whereas a bunch of protein complexes or lipids might be involved as well. However, there is still a lack of ample evidence that fully illustrates the underlying mechanism of the RNA sorting process, and further investigations are deserved.

### **Hypoxia alters exosome release and exosomal components**

**Fig. 1:**
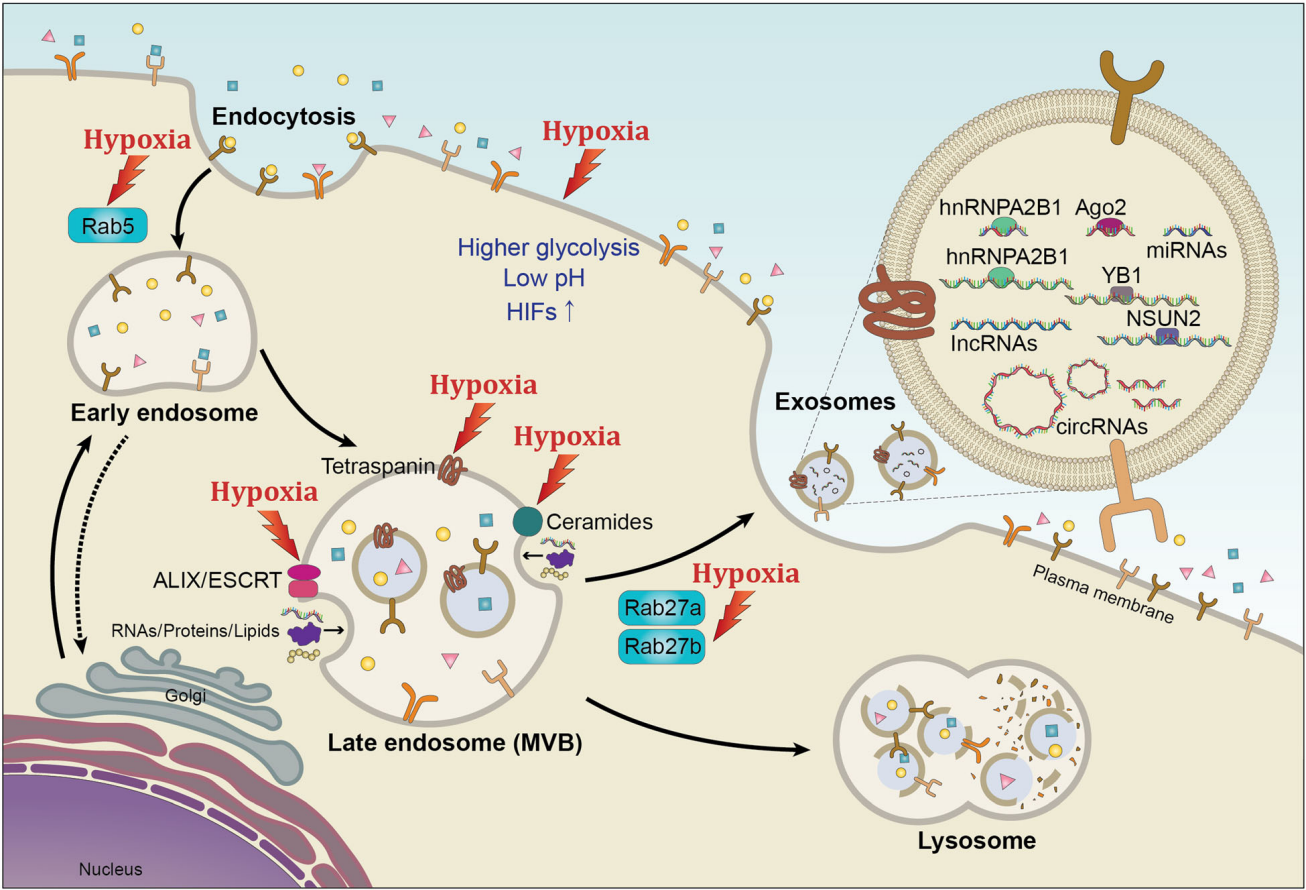
**Hypoxia influences the secretion and non-coding RNA cargos of exosomes**. Legend: Extracellular components enter cells through endocytosis along with the plasma membrane, leading to the formation of early endosomes and late endosomes (MVBs). Some molecules like ESCRT machinery, ALIX, tetraspanins, and ceramides are involved in this process. Several Rab GTPases are associated with MVBs transporting to the plasma membrane. Then, exosomes with specific cargos are released through exocytosis. Hypoxia triggers the alteration in gene expression of HIFs or other signaling pathways, which may impact exosome biogenesis and cargo sorting by regulating these molecules. Besides, non-coding RNAs binding with some RNA binding proteins like hnRNPA2B1, YB1, NSNU2, or Ago2 might be favorably sorted into exosomes

Hypoxia can activate various pathways to promote the secretion of exosomes and alter the components loaded in exosomes (Fig. 1). Rab GTPases such as Rab27a and Rab27b are essential for exosomal secretion pathways in cancer cells [64]. In hypoxic conditions, altered protein expression of small GTPase in cells promotes endocytosis, consequently changing the degree of exosomal secretion [65]. In ovarian cancer, hypoxia significantly promoted exosome secretion through the upregulation of Rab27a. These hypoxia-induced exosomes elicited a more aggressive and chemoresistant phenotype of cancer cells [66]. King et al. also confirmed that hypoxic breast cancer cells produced a higher level of exosomes in a HIF-1α-dependent manner [67]. Rab5 could regulate vesicle-mediated transportation from the cell membrane to early endosomes and early endosome fusion [68]. Under hypoxia, Rab5 was clustered in the perinuclear region while CD63 showed higher co-localization with the actin cytoskeleton of prostate cancer cells, suggesting that hypoxia could enhance exosome secretion through promoting early endosome formation and fusion of multivesicular endosomes with the plasma membrane [69].

The hypoxic conditions not only boost the release of exosomes but also influence the molecules contained within exosomes. Hypoxia-induced exosomes exhibit different patterns of various molecules depending on origin cells and environmental factors. In highly malignant brain tumor glioblastoma multiforme (GBM), hypoxic cancer cell-derived exosomes showed enrichment in hypoxia-associated mRNAs and proteins (e.g., matrix metalloproteinases, IL-8, platelet-derived growth factor (PDGF), caveolin 1, and lysyl oxidase) and activated vascular cells in a hypoxia-dependent mode during cancer progression [70]. Recent studies have shed light on the exosomal non-coding RNA expression and function shift in the hypoxic tumor microenvironment. Especially with the development of sequencing technology, more and more significant non-coding RNAs are being uncovered. It has been reported that miR-21, miR-23a, and lncRNA-UCA1 were upregulated in the exosomes derived from hypoxic cancer cells and promoted cancer progression in various signaling pathways [71–73]. Hypoxia was also reported to regulate the expression of circRNAs. Boeckel et al. first identified that cZNF292, cAFF1, and cDENND4C were upregulated while cTHSD1 was under the hypoxic condition in endothelial cells. Moreover, cZNF292 exhibited proangiogenic activities in vitro [74]. While in cancer, hypoxia could induce the expression of circDENND2A, which could promote the migration and invasion of glioma cells through sponging miR-625-5p [75]. The expression of circHIPK3 was reported to be upregulated in hypoxic exosomes compared with normoxic exosomes derived from cardiomyocytes. It could regulate the oxidative damage in cardiac microvascular endothelial cells by inducing miR-29a/IGF-1 signaling pathway [76]. This is the only report by now focusing on circRNAs in hypoxic exosomes. There is no direct evidence showing the role of hypoxic exosomal circRNAs in cancer yet. Collectively, non-coding RNAs have been reported to exert various functions in translation, RNA splicing, DNA replication, etc. [77–80]. Further investigations are warranted to comprehensively understand the role of non-coding RNAs in the tumor microenvironment.

## Effects of hypoxic exosomal non-coding RNAs in the tumor microenvironment

**Fig. 2:**
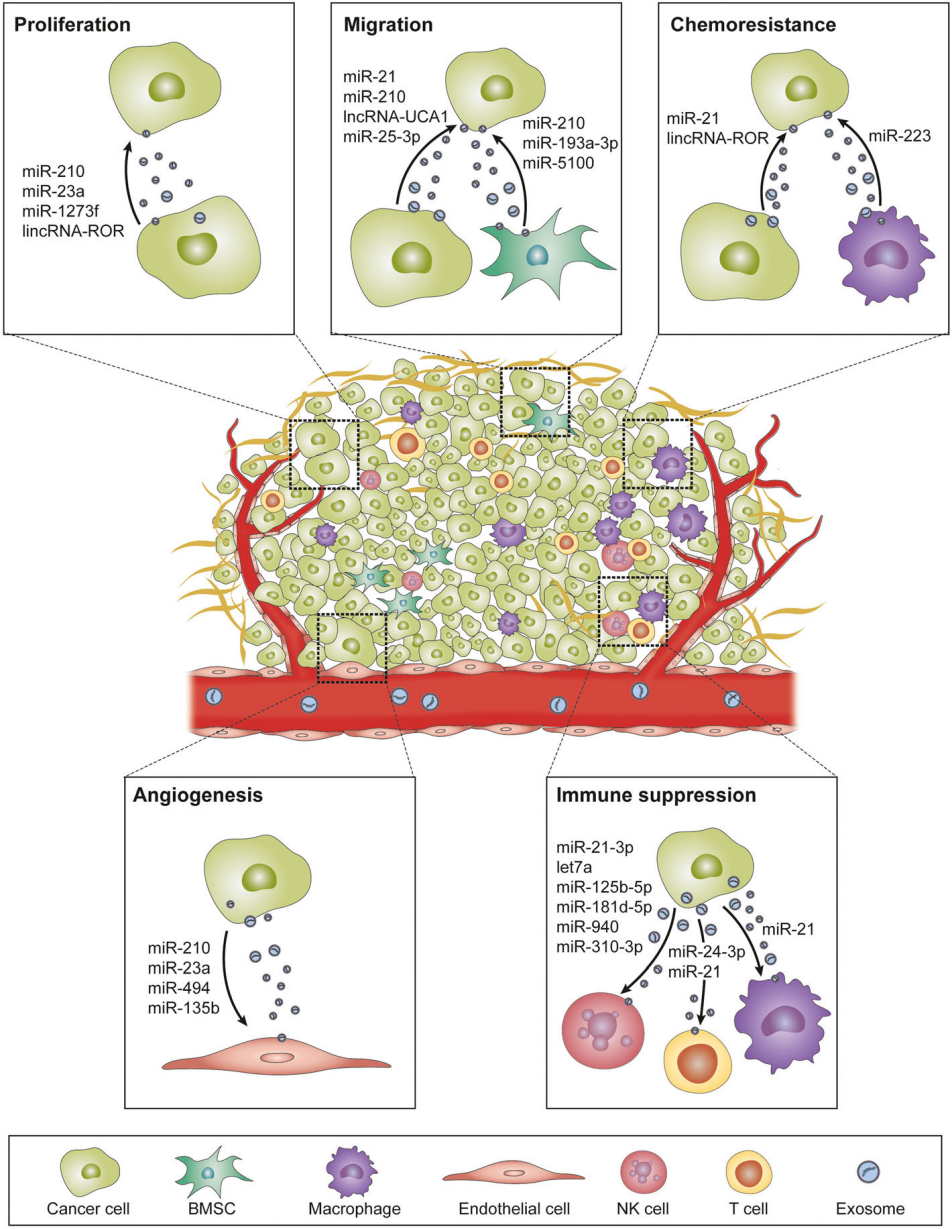
**Exosomal non-coding RNAs regulate the hypoxic tumor microenvironment**. Legend: Hypoxic donor cells impact recipient cells by transmitting non-coding RNAs via exosomes. These exosomal non-coding RNAs can be uptaken by recipient cells and alter their biological behaviors through various pathways, thus regulating tumor development

Exosomes derived from hypoxic cancer cells or stromal cells play a fundamental role in the tumor microenvironment through transmitting non-coding RNAs. Hypoxia-induced exosomal non-coding RNAs have been demonstrated to regulate cancer proliferation, metastasis, chemoresistance, immune response, and angiogenesis, thus reshaping the microenvironment (Fig. 2; Table 1).

**Table 1:** **Non-coding RNAs shuttled via exosomes in the hypoxic tumor microenvironment**

Non-coding RNA	Cancer type	Exosome isolation method	NcRNA identification method	Donor cell	Recipient cell	Function	Mechanism	Ref.
miR-21	glioma	ultracentrifugation	RNA-sequencing	cancer cell	MDSC	promote MDSC expansion and activation	regulating PTEN/PI3K/AKT axis	[81]
	oral squamous cell carcinoma	commercial kit	RNA-sequencing	cancer cell	γδT-cell	stimulate γδT-cell expansion and function	inducing PD-L1 expression though targeting PTEN	[82]
	oral squamous cell carcinoma	commercial kit	qRT-PCR	cancer cell	cancer cell	promote migration and invasion	inducing EMT	[72]
	lung cancer	ultracentrifugation	qRT-PCR	cancer cell	cancer cell	promote chemoresistance	downregulating PTEN and PI3K/ATK pathway	[83]
miR-21-3p	ovarian cancer	commercial kit	miRNA microarray	cancer cell	macrophage	induce M2 polarization	regulating SOCS4/5/STAT3 pathway	[84]
miR-210	leukemia	commercial kit	miRNA microarray	cancer cell	endothelial cell	promote angiogenesis	NA	[37]
	breast cancer	ultracentrifugation& commercial kit	qRT-PCR	cancer stem cell	cancer cell	promote proliferation, Invasion and self-renewal ability	targeting E-cadherin	[67, 85]
	lung cancer	commercial kit	miRNA microarray	BMSC	cancer cell	promote metastasis	inducing STAT3 driven EMT	[86]
miR-23a	liver cancer	ultracentrifugation	qRT-PCR	cancer cell	endothelial cell	promote angiogenesis	targeting SIRT-1	[97]
	lung cancer	commercial kit	qRT-PCR	cancer cell	endothelial cell	promote angiogenesis and increase vascular permeability	inhibiting PHD1 and PHD2 expression, thus enhancing HIF-1α signaling and inhibiting tight junction protein ZO-1	[71]
	lung cancer	ultracentrifugation	miRNA microarray	cancer cell	NK cell	impair NK cell cytotoxicity and NK cell function	targeting CD107a	[35]
lncRNA UCA1	bladder cancer	commercial kit	qRT-PCR	cancer cell	cancer cell	promote migration and invasion, increase tumor growth and progression	inducing EMT	[73]
miR-25-3p	breast cancer	commercial kit	qRT-PCR	cancer cell	cancer cell &macrophage	promote proliferation and migration	stimulating IL-6 secretion from macrophages *via* NF-κB pathway	[88]
miR10a	glioma	ultracentrifugation	RNA-sequencing	cancer cell	MDSC	promote MDSC expansion and activation	regulating RORA/IκBα/NF-κB axis	[81]
miR-1273f	hepatocellular carcinoma	ultracentrifugation	qRT-PCR	cancer cell	cancer cell	promote proliferation	inhibiting LHX6/Wnt/β-catenin pathway	[89]
linc-RoR	hepatocellular carcinoma	commercial kit	qRT-PCR	cancer cell	cancer cell	promote proliferation and increase chemoresistance	Inducing phosphorylation of p70S6K1 (RPS6KB1), PDK1 and HIF-1α protein expression and decreasing miR-145	[90, 91]
miR-193a-3p	lung cancer	commercial kit	miRNA microarray	BMSC	cancer cell	promote metastasis	inducing STAT3 driven EMT	[86]
miR-5100	lung cancer	commercial kit	miRNA microarray	BMSC	cancer cell	promote metastasis	inducing STAT3 driven EMT	[86]
miR-494	lung cancer	ultracentrifugation	qRT-PCR	cancer cell	endothelial cell	promote angiogenesis	downregulating PTEN and activating Akt/eNOS pathway	[92]
let7a	melanoma	ultracentrifugation	qRT-PCR	cancer cell	macrophage	induce M2 polarization of infiltrating myeloid cells and enhance mitochondrial OXPHOS	downregulating insulin-AKT-mTOR signaling pathway	[93]
miR-135b	multiple myeloma	commercial kit	miRNA microarray	cancer cell	endothelial cell	promote angiogenesis	targeting FIH	[94]
miR-24-3p	nasopharyngeal carcinoma	ultracentrifugation	miRNA Microarray	cancer cell	T-cell	inhibit T cell proliferation and differentiation	repressing FGF11, up-regulating p-ERK, p-STAT1, p-STA3, down-regulating p-STAT5	[95]
miR-125b-5p	ovarian cancer	commercial kit	miRNA Microarray	cancer cell	macrophage	induce M2 polarization	regulating SOCS4/5/STAT3 pathway	[84]
miR-181d-5p	ovarian cancer	commercial kit	miRNA Microarray	cancer cell	macrophage	induce M2 polarization	regulating SOCS4/5/STAT3 pathway	[84]
miR-940	ovarian cancer	commercial kit	qRT-PCR	cancer cell	macrophage	induce M2 polarization of MDSC	NA	[36]
miR-223	ovarian cancer	commercial kit	qRT-PCR	macrophage	cancer cell	promote drug resistance	inactivating PI3K/AKT pathway through targeting PTEN	[96]
miR-301a-3p	pancreatic cancer	commercial kit	qRT-PCR	cancer cell	macrophage	induce M2 polarization	downregulating PTEN expression and activating PI3Kγ signaling pathway	[97]

### **Proliferation**

Hypoxia alters tumor metabolism and transcription such as a shift to glycolysis and self-sufficient release of growth signals [98]. Even though much has been known about hypoxia-secreted metabolites promote tumor growth, the importance of hypoxic exosome-mediated tumor growth has been recently grown. Accumulating evidence indicates that pro-tumorigenic molecules secreted through exosomes in the hypoxic tumor microenvironment can promote tumor cell survival and proliferation.

MiR-210 is a well-recognized hypoxia-induced miRNA involved in various biological processes of cancer progression. It was reported to be upregulated in many types of solid tumors and related to unfavorable clinical outcomes of patients [99]. In breast cancer, miR-210 was significantly elevated in the exosomes derived from hypoxic cancer cells than those from normoxic ones [67]. Tang et al. utilized a breast cancer cell spheroid culture model to enrich highly malignant breast cancer stem cells (BCSCs). They corroborated that miR-210 was remarkably upregulated in hypoxic spheroid cells and spheroid-derived BCSCs compared to parental cells. The upregulation of miR-210 promoted the proliferation, self-renewal, and migration of BCSCs [85]. Furthermore, Yu et al. reported that miR-1273f upregulated in hypoxic tumor-derived exosomes promoted cancer proliferation of hepatocellular carcinoma (HCC) by inhibiting LHX6/Wnt/β-catenin pathway [89]. In another research of HCC, Patel and his colleagues showed that hypoxic tumor-derived exosomes reduced cancer cell viability with the increased expression of lncRNA-RoR. Knockdown of lncRNA-ROR induced expression of its target, miR-145, thus decreasing p70S6K1 (RPS6KB1) phosphorylation, PDK1, and HIF-1α expression [90]. Wozniak et al. identified a set of differentially expressed exosomal miRNAs in hypoxic conditions. Hypoxia upregulated miR-494-5p, miR-4497, miR-513a-5p, and miR-6087 while downregulating miR-125b-5p, miR-21-5p, and miR-3934-5p in the exosomes from patient-derived melanoma cell lines cultured under hypoxia. Pathway analysis with bioinformatical tools has shown that these miRNAs were closely associated with tumor survival, but no further experimental validation was carried out [100].

Therefore, exosome-mediated communication plays an essential role in the hypoxic environment. Hypoxic exosome-shuttled bioactive non-coding RNAs have been shown as critical regulators of cancer proliferation.

### **Invasion and metastasis**

Hypoxia has been demonstrated to regulate the invasion and migration ability of cancer cells mainly by promoting EMT. EMT is involved in carcinogenesis and endows transformative properties to cancer cells by improving mobility, invasion, and migration [101]. During EMT, downregulation of epithelial markers (E-cadherin and β-catenin) and upregulation of mesenchymal markers (N-cadherin and vimentin) can occur, which then induce the mesenchymal phenotypes and enhance the metastatic ability of the cancer cells. Much attention has been drawn to exosomal non-coding RNAs in the hypoxic tumor microenvironment, given that they could govern metastatic and invasive capability of cancer cells by directly or indirectly targeting EMT markers. Li et al. reported that miR-21 increased in hypoxia-derived exosome promoted invasion and migration in oral squamous cell carcinoma (OSCC) by inducing EMT [72]. In addition, lncRNA-UCA1 was present at a high level in the hypoxic exosomes from cancer cells than normoxic exosomes. The lncRNA-UCA1 secreted by hypoxic cancer cells promoted tumor progression through upregulating EMT in vivo and in vitro in bladder cancer [73]. Exosomal miR-25-3p released from hypoxic breast cancer cells stimulated migration and proliferation of tumor cells by inducing IL-6 secretion and activating NF-κB signaling in macrophages. In vivo experiments revealed that injection of breast cancer cells with the miR-25-3p inhibitor substantially reduced the tumor size by inhibiting IL-6-mediated STAT3 activation [88].

Stromal cells are also indispensable components in the tumor microenvironment. Stromal-cancer or stromal-stromal interactions mediated by exosomes have profound impacts on metastasis initiation and cancer progression. Zhang et al. demonstrated that cancer cells could uptake exosomes derived from hypoxic bone marrow-derived mesenchymal stem cells (BMSCs) and acquire higher invasiveness. During this process, miR-193a-3p, miR-210-3p, and miR-5100 transferred into cancer cells and activated STAT3 signaling, thus inducing EMT in lung cancer cells. Furthermore, these three miRNAs were upregulated in the plasma-derived exosomes of patients than non-metastatic patients [86].

Metastatic colonization is profoundly affected by the hypoxic tumor microenvironment. Tumor-derived exosomes can reflect the hypoxic characteristics of the originating cell and also are capable of facilitating pre-metastatic niche formation by transmitting non-coding RNAs. Notably, organ-specific metastasis has gained much attention in recent years. Despite the hard work of many scientists, still little has been known about the mechanisms. Exploring exosomal non-coding RNAs could give rise to promising therapeutic targets as well as prognostic biomarkers of cancer metastasis.

### **Chemoresistance**

Resistance to anti-cancer therapies is one of the biggest obstacles in cancer treatment. Intrinsic resistance arises because of genetic alterations, whereas extrinsic resistance due to interactions between tumor cells and microenvironment against chemotherapy. Hypoxia affects the overall course of the tumor including responses to therapies. Hypoxia-induced malignancies could form resistance to various anti-cancer drugs including cisplatin, doxorubicin, sorafenib, etoposide, paclitaxel, and gemcitabine [102]. Also, researches have shown that anti-cancer drug resistance can be restored through inhibition of hypoxia-induced signaling pathway. Hypoxia could regulate chemoresistance of cancer cells by controlling cell cycle, autophagy, senescence of cells, and chemotherapeutic efficacy through acidity as well as drug efflux pump expression [103]. Hypoxia-induced intracellular substances such as proteins and non-coding RNAs via exosomes secretion affect tumor microenvironment and cancer cell chemoresistance.

Dong et al. demonstrated that hypoxic non-small-cell lung cancer (NSCLC) cell-derived exosomes enhanced cisplatin resistance of normoxic cancer cells through transmitting miR-21. Mechanically, miR-21 in hypoxic exosomes downregulated phosphatase and tensin homolog (PTEN) and PI3K/ATK pathway sequentially, triggering cisplatin resistance in normoxic cancer cells. Further analysis with the TCGA database showed that miR-21 expression was positively correlated with HIF-1α and high miR-21 expression indicated worse survival in patients undergoing chemotherapy [83]. Patel and his colleagues found linc-RoR, a stress-responsive lncRNA, regulated chemosensitivity of HCC cells to sorafenib, and doxorubicin. They described that linc-ROR expression was enhanced in the hypoxic cancer cell-derived exosomes. TGF-β enriched linc-RoR within exosomes, resulting in suppression of chemotherapy-induced cell death and tumor-initiating cell proliferation [91].

It is noteworthy that chemoresistance does not arise intrinsically in cancer cells. Many stromal cells like CAFs also support cancer cells to gain resistance to chemotherapy. MiR-223 was demonstrated to promote proliferation and invasiveness of ovarian cancer cells by targeting SOX11 expression [104], and it was the most significantly upregulated miRNA in the recurrent serous ovarian cancer tissues as compared to the primary tissues [105]. On this basis, Zhu et al. observed the upsurge of miR-223 in TAMs and TAM-derived exosomes under hypoxia. MiR-223 conveyed by hypoxic exosomes could reduce apoptosis, increase cell viability, and also increase drug resistance of ovarian cancer cells via downregulating PTEN expression and activating PI3K/AKT signaling pathway. Moreover, higher miR-223 expression was shown in the cisplatin-resistant patients and recurrent patients in the epithelial ovarian cancer (EOC) patient-derived specimens. High expression of miR-223 together with low expression of PTEN indicated a bad prognosis of ovarian cancer patients [96].

These studies indicated chemoresistance could be passed through transmitting non-coding RNAs in exosomes in the hypoxic microenvironment. Blocking chemoresistance-associated exosomal non-coding RNAs could be a promising way to overcome chemoresistance.

### **Immune suppression**

In principle, tumor development can be monitored by cytotoxic innate and adaptive immune cells. However, as the tumor develops from dysplasia to clinically detectable tumors, cancer cells evolve different mechanisms such as losing expression of tumor antigens or modifying immune checkpoint molecules in order to avoid the destruction by the immune system [106]. Tumor-derived exosomes have been shown to induce tumor-specific or nonspecific immune responses. Antigen-presenting cells could uptake tumor-associated antigens shuttled in exosomes and stimulate the tumor suppressive reactions [107]. Hypoxic tumor exosomes have been demonstrated to suppress T cell proliferation and NK cell activation, induce macrophage M2 polarization and regulatory T cells (Tregs) activation, and increase MDSC population resulting in immune dysfunction.

The interactions between tumor cells and T cells are critical in tumor immune microenvironment. Tumoral exosomes carrying biologically active non-coding RNAs transmit signals between cells, promoting tumor progression and immune escape. Ye et al. first reported that nasopharyngeal carcinoma (NPC) cell-derived exosomes impeded T cell dysfunction through differential miRNA expression [108]. Building on this study, they further identified that tumoral exosomes could hamper T cell function by miR-24-3p delivery. Mechanistically, hypoxia-induced exosomal miR-24-3p inhibited T cell proliferation and Th1, Th17 differentiation and inducing Treg differentiation by targeting FGF11 followed by the upregulation of p-ERK, p-STAT1, p-STAT3, and downregulation of p-STAT5 [95].

T cell exhaustion is characterized as the upregulation of PD-1 on T cells. Blocking the PD-1/PD-L1 axis is the best way to reinvigorate T cell function [109]. γδ T cell is a unique lymphocyte population that has been reported to have either anti- or pro-tumoral functions. Li et al. revealed that normoxic OSCC-derived exosomes stimulated the γδ T cell expansion and cytotoxic function in a dendritic cell (DC)-independent manner, which could be attenuated by miR-21-enriched hypoxic exosomes. MDSCs are a heterogeneous immunosuppressive cell population from myeloid lineage migrating to the tumor site through circulation [110]. A chronic inflammatory environment rich in cytokines like TNF, TGF-β, and IL-10 triggers myeloid cells converting to MDSCs. Therefore, they are normally present in cancer or chronic inflammation-associated diseases while not present in a steady state of healthy people [111]. MDSCs suppress the adaptive and innate immunity through inhibiting T cell activation, promoting M2 polarization of macrophages, inducing CAF differentiation and inhibiting NK cell cytotoxicity, thus contributing to tumor angiogenesis, metastasis, and drug resistance [112]. The accumulation of MDSCs was shown to be correlated with poor clinical outcomes and reduce the efficacy of immunotherapy in cancer patients. Hence, eliminating and suppressing MDSCs is becoming a new therapeutic strategy [113]. It was reported that exosome-shuttled miR-21 secreted from γδ T cells could abate the function of MDSCs in a PD-L1-dependent manner through targeting PTEN [82]. This study sheds new light on the effects of tumor-derived exosomes on the γδ T cells. Meanwhile, it also suggested that integrative inhibition of hypoxia-induced exosomal miRNAs and PD-1/PD-L1 axis would be a new insight in immunotherapy. In glioma, hypoxia-induced exosomal miR-21 and miR-10a presented the remarkable effects on the expansion and function of MDSCs in vitro and in vivo via miR-21/PTEN/PI3K/AKT and miR-10a/RORA/IκBα/NF-κB axis respectively [81].

Hypoxia in the tumor microenvironment could stimulate immunosuppressive effects attenuating cytotoxic T lymphocyte (CTL) and NK cell-mediated tumor cell lysis. Berchem et al. elucidated that lung cancer cells generated exosomes with higher miR-23a expression, which could impair NK cell cytotoxicity and NK cell function by directly targeting CD107a. Furthermore, higher TGF-β in the exosomes might partly contribute to the enrichment of miR-23a. This is the first study demonstrating how cancer cells in the hypoxic microenvironment educate NK cells through exosome transmitted non-coding RNAs [35].

Macrophages can be roughly classified into M1 (classically activated) macrophages with pro-inflammatory effects and M2 (alternatively activated) macrophages with anti-inflammatory effects. M1 macrophages characterized by the expression of the inducible type of nitric oxide synthase (iNOS) are proinflammatory, whereas M2 macrophages express high level of anti-inflammatory cytokines (e.g., IL10) and a potent arginase-1 (Arg1) activity to favor tumor cell growth [114]. TAMs constitute a predominant population of immune-related stromal cells in the tumor microenvironment. TAMs were previously described as exhibiting M2-like function. However, studies argue that TAMs can express hallmarks of both M1 and M2 polarization [115]. Cancer cells under hypoxia pressure secreted more functional exosomes acted as a bridge between cancer cells and macrophages, especially inducing M2 polarization. Although M2 polarization is still the mainstream, altering TAMs to a predominantly M1 phenotype has been put on the agenda for developing new immunotherapeutic strategies. These results suggest that hypoxic exosomes are influential elements in the tumor microenvironment mediating the interactions between cancer cells and macrophages.

Hypoxic EOC cell-derived exosomes containing high levels of miR-940 stimulated the M2 polarization of macrophages. Then, miR-940-induced M2 macrophages, in turn, promoted EOC cell proliferation and migration. Interestingly, a high level of miR-940 was also observed in the malignant ascites-derived exosomes compared with benign peritoneal fluids [36]. The same research team further explored the different miRNA expression profiling patterns between cancer cell-derived exosomes under hypoxia and normoxia. MiR-21–3p, miR-125 b-5p and miR-181 d-5p were selected as HIF-1α and HIF-2α induced miRNA candidates and then validated to affect macrophage M2 polarization through regulating SOCS4/5/STAT3 pathway [84]. Moreover, exosomal miR-301a-3p derived from hypoxic pancreatic cancer cells promoted macrophage M2 polarization through downregulation of PTEN expression and activating PI3Kγ signaling pathway, triggering the secretion of TGF-β, IL-10, and arginase from pancreatic cancer cells, which in turn facilitated EMT and lung metastasis [97]. Quantitative proteomics revealed that hypoxic mouse melanoma cell-derived exosomes were rich in immunomodulatory proteins and chemokines including CSF-1, CCL2, FTH, FTL, and TGFβ. Meanwhile, they detected that miRNA let7a was decreased in the hypoxic cells but dramatically increased in the released exosomes. Let7a-loaded exosomes enhanced mitochondrial oxidative phosphorylation system (OXPHOS) in macrophages through suppressing the insulin-AKT-mTOR signaling pathway [93].

Hypoxia pressure promoted the production and secretion of immune-modulating exosomes rich in non-coding RNAs. Exosome-mediated cancer-immune cell communication substantially impacts the immune reaction. Immune checkpoints are accessory molecules with T cell activation or inhibition effects. Blockage of cytotoxic T lymphocyte antigen 4 (CTLA-4) and PD-1 have heralded the dawn of an immune therapy era. Exosomal non-coding RNAs might be the indispensable targets for immune therapy based on their immune regulating functions.

### **Angiogenesis**

Angiogenesis refers to a new blood vessel-forming process and is an essential mediator for tumor progression. Hypoxia is one of the key factors in tumor angiogenesis. Vascular immaturity and weakened cell association of tumor blood vessel network can lead to excessive permeability, poor perfusion, and increased hypoxia [116]. Angiogenesis is a complicated process comprising of many genes, regulators, and pathways. VEGF is the crucial pro-angiogenetic growth factor stimulated by the alarm of hypoxia. Carmeliet et al. found that the hypoxic induction of VEGF decreased significantly in mouse embryonic stem cells by inhibiting HIF-1α [117]. In addition to VEGF, HIF-1α is capable of regulating some other angiogenesis-associated factors like platelet-derived growth factor (PDGF-B), vascular endothelial growth factor receptor-1 (VEGFR-1), endothelin-1, iNOS, and epidermal growth factor (EGF).

Recent studies have demonstrated that exosomal non-coding RNAs under hypoxic conditions could contribute to the angiogenesis process. For instance, miR-210 upregulated both in hypoxic leukemia cells and exosomes enhanced tube formation in endothelial cells [37]. In NSCLC, HIF-1α-induced microvascular miR-494 promoted angiogenesis both in vitro and in vivo through downregulating PTEN and activating Akt/eNOS pathway [92]. Another study in lung cancer showed that hypoxic exosomes promoted angiogenesis through transmitting miR-23a. Knockdown of HIF-1α prevented the increase of exosomal miR-23a while miR-23a inhibited HIF-1α regulators, PHD1, and PHD2 expression and enhanced HIF-1α signaling. Meanwhile, hypoxia-induced exosomal miR-23a could inhibit tight junction protein ZO-1 and increase vascular permeability [71]. Interestingly, exosomal miR-23a upregulation was also observed in chemical-induced hypoxic liver cancer cell colonies established from culturing hepatic cancer cells on the soft agar. Results suggested that exosome-shuttled miR23a was capable of inducing angiogenesis by regulating pro-angiogenic marker genes and targeting SIRT-1 in chick chorioallantoic membrane (CAM), in ovo xenograft, and in HUVEC model system [87]. Umezu et al. established hypoxia-resistant multiple myeloma cell lines under 6 to 7 months hypoxic incubation to better mimic in vivo conditions of hypoxia bone marrow. Exosomal miR-135b derived from those cell lines enhanced endothelial tube formation under hypoxia via the HIF-FIH signaling pathway. Current studies mainly focused on acute hypoxia, which may differ from in vivo conditions. This is the first study that reported exosome-mediated cell-cell communication under chronic hypoxia [94].

Hypoxic exosomal non-coding RNAs can regulate angiogenesis by inducing phenotypic and functional changes in endothelial cells to promote tumor growth and metastasis. VEGFR inhibitors serve as the most vastly applied anti-angiogenesis agents now. They are bringing beneficial efficacies as well as nonnegligible problems like a double-edge sword. Exploiting the anti-angiogenesis potential of RNA therapy via exosomes might be a new therapeutic strategy in the future.

## Clinical applications

**Fig. 3:**
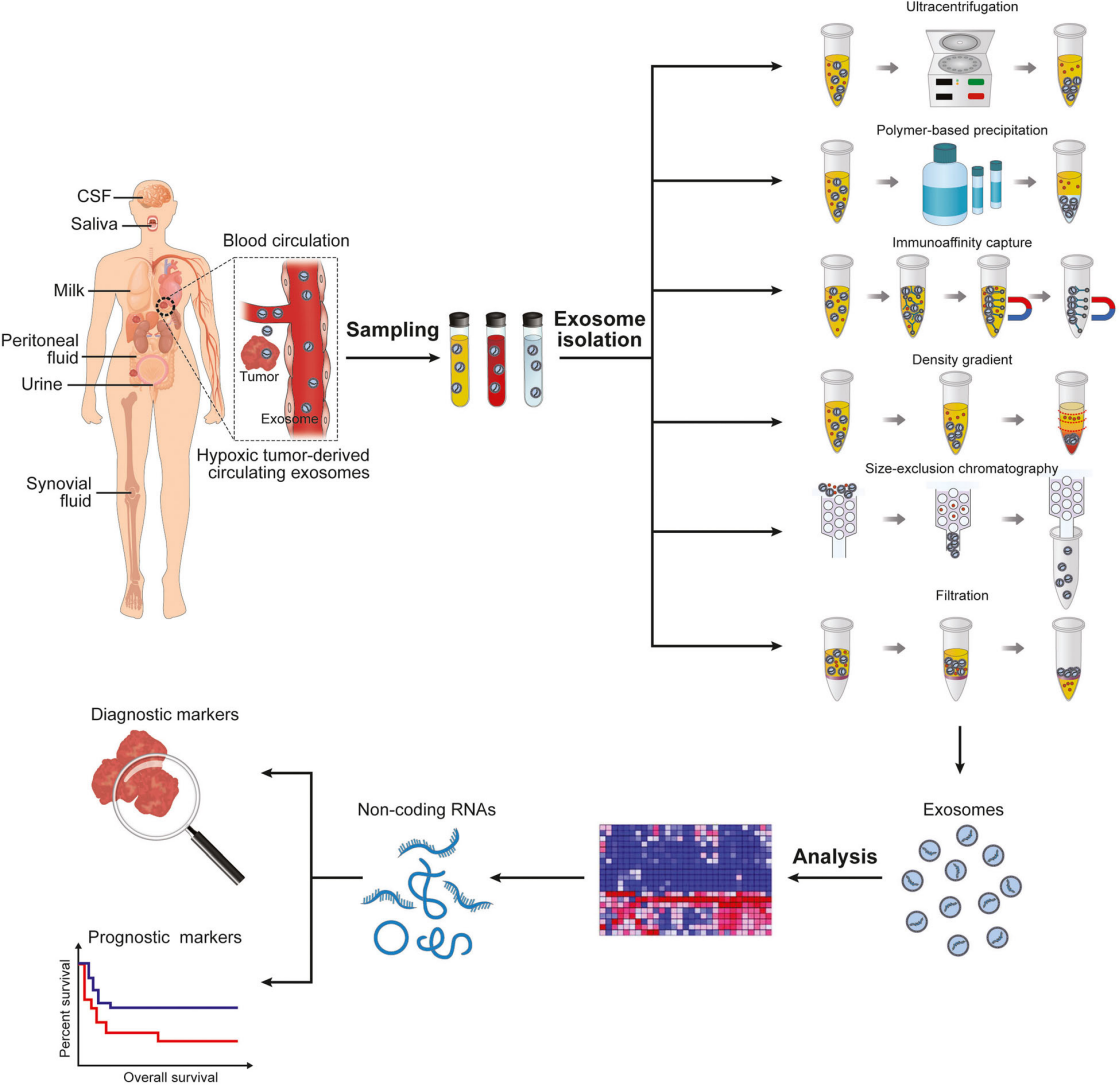
**Hypoxic tumor-derived circulating exosomal non-coding RNAs in liquid biopsy**. Legend: Exosomes are present in diverse biofluids including blood, cerebrospinal fluids, saliva, milk, peritoneal fluid, urine, and synovial fluid. Exosomes can be isolated from these biofluids, and non-coding RNAs are then analyzed for diagnostic or prognosis markers. As illustrated on the right side of the figure, various methods have been developed to isolate exosomes with different advantages and disadvantages each (ultracentrifugation: high sample capacity, minor impacts on exosomal components; time consuming, facility dependent. Polymer-based precipitation reagents: simple steps, possible for small sample volume, high yield; expensive reagents, low purity; immunoaffinity capture: high purity and specificity; expensive reagent, low yield, antibody dependent; density gradient separation: high purity; complicated procedures, low yield, facility dependent. Ultrafiltration: less time consuming; low purity and integrity)

Liquid biopsy is a new diagnostic tool performed on blood or other biofluids to assess the tumor-derived components and their genomic or proteomic profiles [118, 119]. Circulating tumor cells (CTCs) and circulating tumor DNAs (ctDNAs) are the primary analytes of liquid biopsies. However, recent studies have uncovered more liquid biopsy analytes including tumor-educated platelets, circulating non-coding RNAs, and tumor-derived exosomes [120]. Exosomes are released by almost all cell types and have been detected in various biofluids, including blood, urine, saliva, ascites, and cerebrospinal fluids. Exosomes and non-coding RNAs shuttled in exosomes have been demonstrated to play a vital role in cancer progression. Hypoxia is one of the critical characteristics in the microenvironment of most solid tumors. Analyzing exosomes or exosomal non-coding RNAs in the biofluids could monitor cancer progression, predict drug resistance, even detect tumor heterogeneity and trace tumor evolution, contributing to the precision therapy of cancer patients (Fig. 3).

In vitro studies have shown that hypoxia could promote the production and release of exosomes of various cancer cell lines [67, 71, 87, 94]. As a consequence, hypoxic tumors may increase the concentration of exosomes in the blood or other biofluids. Osti et al. compared the concentration of plasma exosomes in patients with GBM and healthy controls where GBM patients showed significantly higher concentration. Intriguingly, the concentration decreased to an almost similar level to healthy controls after surgery while an increase was detected again at recurrence. This indicated that tumor cells mainly contributed to the exosome increment in plasma [121]. Likewise, higher levels of exosomes were detected in the plasma of NPC patients than healthy controls and positively correlated with tumor lymph node metastasis and shorter disease-free survival (DFS) [108]. These data suggested that circulating exosome enumeration is applicable to describe disease status and predict the clinical outcomes of patients.

However, the exosome releasing and producing processes are complicated and multifaceted. Hypoxia is not the sole factor influencing quantity and quality of exosomes. Besides, the mechanism of hypoxia in vivo might be quite different with that of in vitro models. Some other factors may also stimulate the release of exosomes including thermal stress, oxidative stress, tumor pH value, autophagy, endoplasmic reticulum stress, increase in intracellular Ca^2+^ levels, or drug intervention [122]. Therefore, circulating exosome quantification has many limitations due to its non-specificity. Analyzing exosome contents is needed to evaluate the disease accurately.

Non-coding RNAs can be transferred through exosomes from tumor cells to adjacent cells or remote organs by entering the blood circulation. These non-coding RNAs are protected from degradation and stably exist in the circulation. Thus, exosomal non-coding RNAs derived from hypoxic tumors are detectable in the blood and might be used as biomarkers of cancer progression (Table 2). Exosomes in the biofluids are a heterogeneous population of different cell origins. It is important to find tumor-specific non-coding RNA biomarkers. Recent studies have demonstrated different non-coding RNA profiles in the exosomes derived from cancer patients and healthy people through RNA-sequencing or RT-PCR analysis [53]. Differently expressed non-coding RNAs generated from those studies could provide a large number of possible candidate biomarkers. By comparing exosomal non-coding RNA expression patterns at baseline, pre-treatment, and post-treatment, correlating with clinical parameters, combining with continuous follow-up, it is possible to predict patient prognosis and therapeutic response. The combination of several non-coding RNA markers together may have favorable sensitivity and specificity than a single non-coding RNA. Wang et al. reported that the area under the ROC curve of dual detection of exosomal HOTAIR and miR-21 for diagnosing laryngeal squamous cell carcinoma reached 87.6%, significantly higher than 80.1% of miR-21 or 72.7% of HOTAIR [124]. In addition, applying non-coding RNA biomarkers could compensate for the deficiency of clinical protein markers. For example, prostate-specific antigen (PSA) used for screening prostate cancer is of high sensitivity but low specificity in that it might also be upregulated in benign hyperplasia. The expression level of exosomal lncRNA SChLAP1 could help to differentiate prostate cancer and benign prostate hyperplasia patients when PSA was moderately elevated [125]. Integrating in silicon analyses or in vitro experiments with the expression of non-coding RNAs in patient-derived samples is capable of selecting significant and functional non-coding RNAs of relative high specificity and sensitivity. In vitro models developed under hypoxic conditions can better mimic the in vivo state of the tumor microenvironment. Thus, hypoxia-induced exosomal non-coding RNAs might be of great significance in cancer diagnosis and monitoring.

**Table 2:** **Hypoxic tumor-derived circulating exosomal non-coding RNAs as diagnostic and prognostic biomarkers**

Cancer type	Sample type	Sample size	Sample grouping	Non-coding RNA	Expression	Clinical significance	Ref.
locally advanced rectal cancer	plasma	24	cancer patients and healthy donors	miR-486-5p	downregulated	associated with organ-invasive primary tumor and lymph node metastasis	[123]
				miR-181a-5p	downregulated	associated with organ-invasive primary tumor and lymph node metastasis	
				miR-30d-5p	upregulated	associated with metastatic progression	
ovarian cancer	serum	12	recurrence patients and primary patients	miR-223	upregulated	associated with recurrence	[96]
nasopharyngeal carcinoma	plasma	85	cancer patients and healthy donors	miR-24-3p	upregulated	negatively associated with DFS	[95]
lung cancer	serum	30	cancer patients and healthy donors	miR-23a	upregulated	diagnostic marker	[71]
OSCC	serum	216	cancer patients and healthy donors	miR-21	upregulated	associated with T stage, and N stage	[72]
Bladder cancer	serum	60	cancer patients and healthy donors	lncRNA-UCA1	upregulated	diagnostic marker	[73]

Hypoxia-induced exosomal miR-23a and miR-24-3p were reported to be markedly higher in the serum of lung cancer patients and NPC patients, respectively, than that of healthy volunteers. Further analysis showed that the serum exosomal miR-24-3p level was negatively associated with the DFS of NPC patients [71, 95]. Likewise, patients with OSCC had much greater expression of circulating exosomal miR-21 compared with paired healthy donors. High circulating exosomal miR-21 levels were closely associated with the T stage and lymph node metastasis [72]. Moreover, serum-derived exosomes of bladder cancer patients showed a significantly higher level of lncRNA UCA1, and notably, the lncRNA UCA1 levels were positively correlated with HIF-1α expression. It could be speculated that intratumoral hypoxia might be capable of boosting exosome-shuttled lncRNA-UCA1expression. ROC analysis also showed fine specificity and sensitivity, suggesting its high diagnostic and clinical significance [73]. In locally advanced rectal cancer (LARC), Tonje et al. filtered a series of exosomal miRNAs from five hypoxic cell lines and validated in patient plasma samples. Among these, downregulated miR-486-5p and miR-181a-5p were correlated with T4 and lymph node metastasis-positive disease, respectively, while upregulated exosomal miR-30d-5p was associated with the metastatic progression [123].

Hypoxia influences not only cancer cells but also stromal cells. Zhang et al. reported that hypoxic BMSC-derived exosomes could promote lung cancer metastasis through transmitting miR-193a-3p, miR-210-3p, and miR-5100. Those three miRNAs expressed remarkably higher in the plasma-derived exosomes of lung cancer, liver cancer, and pancreatic cancer patients than the counterparts of healthy controls. In addition, ROC analysis of the individual miRNA or combination of three miRNAs panel all showed high specificity and sensitivity in distinguishing metastatic lung cancer patients and non-metastatic lung cancer patients [86].

Circulating exosomes can reflect the hypoxic feature of primary tumors. Therefore, non-coding RNAs loaded in circulating exosomes are emerging as new biomarkers of liquid biopsy in that they can dynamically mirror tumor burden or clinical events. As tumor progression and evolution is an intricate process, a single biomarker is far from comprehensively explaining the disease status. Integrative analysis of multiple biomarkers might be a prospective trend for clinical diagnosis. Although these researches have brought out new insights into the diagnostic and prognostic value of hypoxic tumor-derived exosomal non-coding RNAs, there are still some limitations. Studies discussed here had a minimal patient sample size so that large-scale investigations are highly demanded to validate the clinical potential of hypoxic exosomal non-coding RNAs. Hypoxic exosomes can partially reflect the hypoxic feature of solid tumors. Researchers used different methods to induce hypoxia pressure which varied from oxygen concentration, hypoxic incubation time, etc. By far, there are no optimal in vitro systems to precisely mimic the in vivo hypoxic tumor microenvironment. Hypoxic exosome concentration and cargo may fluctuate depending on different hypoxia generating means. Meanwhile, they were using different ways to isolate exosomes, which may cause nonnegligible variations as well. Thus, standardized protocols that can generate high purity of exosomes are undeniably needed for future clinical applications. Furthermore, recent researches preferred to use RNA sequencing or microarray to screen differently expressed non-coding RNAs, which could generate vast sets of candidate non-coding RNAs but at the same time increasing the difficulty of data analysis to a large extent. How to better analyze the data and related pathways also remains a big issue. Notably, which molecule to use to normalize the exosomal lncRNAs or miRNAs is still controversial.

Exosomes are stable, easy-stored, biocompatible, highly permeable, low toxic, and immunogenic, which suffice almost all the merits of a good drug delivery system [126]. Exosomes have the natural encapsulating ability to carry different anticancer drugs, natural agents, and nucleic acid therapeutics including miRNAs, siRNAs, or even gene-editing systems, such as CRISPR-Cas9 system. Notably, miRNAs and siRNAs transferred through exosomes to the recipient cells can partly or entirely abolish the expression of target genes, thus restricting tumor progression [126]. The KRASG^120^ siRNA loaded in human-foreskin fibroblast-derived exosomes efficiently suppressed pancreas desmoplasia in the orthotopic and genetically engineered mice [127]. Despite the advantages over other drug delivery systems, exosomes are still facing unavoidable challenges. Exosome heterogeneity, unwanted immunogenetic possibility, and the potential tumor-promoting risks await resolutions before clinical translation [126].

## Conclusions and prospectives

Exosomes shuttled between different cells and have a crucial role in intercellular communication. Exosomes encapsulate a variety of biologically active molecules, including lipids, proteins, and nucleic acids. In the hypoxic tumor microenvironment, exosomes present an altered pattern of genes and proteins. Hypoxia-induced non-coding RNAs enveloped in exosomes have been demonstrated to be involved in multiple physiological processes including cancer proliferation, pro-metastasis niche formation, chemoresistance, angiogenesis, and immune reactions. However, deeper mechanisms underlying the releasing and cargo machinery in the hypoxic conditions need to be further explored.

Given that hypoxic exosomal non-coding RNAs are determinable in the body fluids, they are emerging to be fine analytes for liquid biopsy. However, numerous obstacles await resolutions before clinical translation. A technical hurdle worth mentioning is how to generate pure and homogenous exosomes. Currently, the most commonly used techniques for isolating exosomes include traditional ultracentrifugation, exosome precipitation reagents, density gradient separation, immunomagnetic beads, and ultrafiltration. However, these methods have various drawbacks in several aspects like specific equipment-dependence, complicated and time-consuming procedures, low productivity, and purity. Therefore, more precise, reliable, and accessible approaches are in strong need for isolation and characterization of exosomes. Recently, some novel exosome isolating methods have come out, such as microfluidic devices, nanoplasmon-enhanced scattering (nPES), membrane-mediated exosome separation, and lab-on-a-chip devices. On the one hand, these methods may simplify the isolation procedures and improve the productivity or purity of exosomes. On the other hand, there is still a lack of laboratory testing experience and preclinical practices [128]. Hopefully, a standardized method for exosome isolation will be developed in the near future. Besides, cancer is a very heterogeneous disease with different gene expression patterns in different sites and biofluids. Chen et al. showed that miRNA profiles were different even in different fractions of human blood, indicating some biomarkers might only be detected in some specific biofluids. Therefore, proper biofluids need to be selected according to the characteristics of different cancer, which should be easily accessible through non-invasive or minimally invasive approaches [129].

Recent studies have shown that gene therapy and immunotherapy manipulating exosomes are no longer just a fantasy, although numerous obstacles await resolutions before clinical translation. Exosomes can be obtained from various cells. However, it is not sure if the cellular origin would affect the therapeutic effects of exosomes. Efforts should be made to find the optimal therapeutic source of exosomes and improve loading efficiency. Another major obstacle following by efficient encapsulation of specific agents like non-coding RNAs is the delivery route. There is still a lack of verification if these modified exosomes should be delivered through local inoculation directly into the tumor sites or by systemic administration. More in-depth investigations are strongly required before clinical translation.

Currently, there are 158 exosome-associated clinical trials registered at https://clinicaltrials.gov/. Among these studies, 17 are not enrolling yet, 70 are active and recruiting, 4 are enrolling by invitation, 20 are active but not enrolling, 26 were completed, 1 was withdrawn, and 16 are in unknown status. Overall, the preclinical and clinical studies are just over the horizon, but the dawn of an exosome era is expected with the indefatigable endeavor of scientists.

## Data Availability

Not applicable.

## Abbreviations

miRNAs: Micro RNAs
lncRNAs: Long non-coding RNAs
miRISC: MiRNA-induced silencing complex
EVs: Extracellular vesicles
ROS: Reactive oxygen species
ECM: Extracellular matrix
HIF-1: Hypoxia-inducible factor-1
PHD: Prolyl hydroxylase
VHL: Von Hippel-Lindau
PD-1: Programmed cell death protein 1
PD-L1: Programmed death-ligand 1
MDSC: Myeloid-derived suppressor cell
CAF: Cancer-associated fibroblast
IGF1: Insulin-like growth factor 1
MVBs: Multi-vesicular bodies
ESCRT: Endosomal-sorting complex required for transport
NK cell: Natural killer cell
PDGF: Platelet-derived growth factor
BMSC: Bone marrow-derived mesenchymal stem cell
TAM: Tumor-associated macrophage
Treg: Regulatory T cell
DC: Dendritic cell
CTLA-4: Cytotoxic T lymphocyte antigen 4
iNOS: Inducible nitric oxide synthase
EGF: Epidermal growth factor
EMT: Epithelial-mesenchymal transition
HREs: Hypoxic response elements
NPC: Nasopharyngeal carcinoma
GBM: Glioblastoma multiforme
EOC: Epithelial ovarian cancer
OSCC: Oral squamous cell carcinoma
HCC: Hepatocellular carcinoma
NSCLC: Non-small-cell lung cancer

## References

[1] Dayan F, Mazure NM, Brahimi-Horn MC, Pouyssegur J (2008). A dialogue between the hypoxia-inducible factor and the tumor microenvironment. Cancer Microenviron.

[2] Waldmann TA (2018). Cytokines in cancer immunotherapy. Cold Spring Harb Perspect Biol.

[3] Cadamuro M, Brivio S, Spirli C, Joplin RE, Strazzabosco M, Fabris L (2017). Autocrine and paracrine mechanisms promoting chemoresistance in cholangiocarcinoma. Int J Mol Sci.

[4] Wegiel B, Vuerich M, Daneshmandi S, Seth P (2018). Metabolic switch in the tumor microenvironment determines immune responses to anti-cancer therapy. Front Oncol.

[5] Jing X, Yang F, Shao C, Wei K, Xie M, Shen H (2019). Role of hypoxia in cancer therapy by regulating the tumor microenvironment. Mol Cancer.

[6] Kuchuk O, Tuccitto A, Citterio D, Huber V, Camisaschi C, Milione M (2018). pH regulators to target the tumor immune microenvironment in human hepatocellular carcinoma. Oncoimmunology..

[7] Lim B, Woodward WA, Wang X, Reuben JM, Ueno NT (2018). Inflammatory breast cancer biology: the tumour microenvironment is key. Nat Rev Cancer.

[8] Weinberg F, Ramnath N, Nagrath D (2019). Reactive oxygen species in the tumor microenvironment: an overview. Cancers (Basel).

[9] Casazza A, Di Conza G, Wenes M, Finisguerra V, Deschoemaeker S, Mazzone M (2014). Tumor stroma: a complexity dictated by the hypoxic tumor microenvironment. Oncogene..

[10] Campbell EJ, Dachs GU, Morrin HR, Davey VC, Robinson BA, Vissers MCM (2019). Activation of the hypoxia pathway in breast cancer tissue and patient survival are inversely associated with tumor ascorbate levels. BMC Cancer.

[11] Erkan M, Kurtoglu M, Kleeff J (2016). The role of hypoxia in pancreatic cancer: a potential therapeutic target?. Expert Rev Gastroenterol Hepatol.

[12] Han Y, Kim B, Cho U, Park IS, Kim SI, Dhanasekaran DN (2019). Mitochondrial fission causes cisplatin resistance under hypoxic conditions via ROS in ovarian cancer cells. Oncogene..

[13] Salem A, Asselin MC, Reymen B, Jackson A, Lambin P, West CML (2018). Targeting hypoxia to improve non-small cell lung cancer outcome. J Natl Cancer Inst.

[14] Yang L, Roberts D, Takhar M, Erho N, Bibby BAS, Thiruthaneeswaran N (2018). Development and validation of a 28-gene hypoxia-related prognostic signature for localized prostate cancer. EBioMedicine..

[15] Petrova V, Annicchiarico-Petruzzelli M, Melino G, Amelio I (2018). The hypoxic tumour microenvironment. Oncogenesis..

[16] Akanji MA, Rotimi D, Adeyemi OS (2019). Hypoxia-inducible factors as an alternative source of treatment strategy for cancer. Oxidative Med Cell Longev.

[17] McKeown SR (2014). Defining normoxia, physoxia and hypoxia in tumours-implications for treatment response. Br J Radiol.

[18] Camuzi D, de Amorim ISS, Ribeiro Pinto LF, Oliveira Trivilin L, Mencalha AL, Soares Lima SC (2019). Regulation is in the air: the relationship between hypoxia and epigenetics in cancer. Cells.

[19] Kang J, Shin SH, Yoon H, Huh J, Shin HW, Chun YS (2018). FIH is an oxygen sensor in ovarian cancer for G9a/GLP-driven epigenetic regulation of metastasis-related genes. Cancer Res.

[20] Li Y, Patel SP, Roszik J, Qin Y (2018). Hypoxia-driven immunosuppressive metabolites in the tumor microenvironment: new approaches for combinational immunotherapy. Front Immunol.

[21] Tawadros AIF, Khalafalla MMM (2018). Expression of programmed death-ligand 1 and hypoxia-inducible factor-1alpha proteins in endometrial carcinoma. J Cancer Res Ther.

[22] Noman MZ, Desantis G, Janji B, Hasmim M, Karray S, Dessen P (2014). PD-L1 is a novel direct target of HIF-1alpha, and its blockade under hypoxia enhanced MDSC-mediated T cell activation. J Exp Med.

[23] Deng J, Li J, Sarde A, Lines JL, Lee YC, Qian DC (2019). Hypoxia-induced VISTA promotes the suppressive function of myeloid-derived suppressor cells in the tumor microenvironment. Cancer Immunol Res.

[24] Daniel SK, Sullivan KM, Labadie KP, Pillarisetty VG (2019). Hypoxia as a barrier to immunotherapy in pancreatic adenocarcinoma. Clin Transl Med.

[25] Kugeratski FG, Atkinson SJ, Neilson LJ, Lilla S, Knight JRP, Serneels J (2019). Hypoxic cancer-associated fibroblasts increase NCBP2-AS2/HIAR to promote endothelial sprouting through enhanced VEGF signaling. Sci Signal.

[26] De Francesco EM, Lappano R, Santolla MF, Marsico S, Caruso A, Maggiolini M (2013). HIF-1alpha/GPER signaling mediates the expression of VEGF induced by hypoxia in breast cancer associated fibroblasts (CAFs). Breast Cancer Res.

[27] Hirakawa T, Yashiro M, Doi Y, Kinoshita H, Morisaki T, Fukuoka T (2016). Pancreatic fibroblasts stimulate the motility of pancreatic cancer cells through IGF1/IGF1R signaling under hypoxia. PLoS One.

[28] Andersen S, Richardsen E, Moi L, Donnem T, Nordby Y, Ness N (2016). Fibroblast miR-210 overexpression is independently associated with clinical failure in prostate cancer - a multicenter (in situ hybridization) study. Sci Rep.

[29] Ela S, Mager I, Breakefield XO, Wood MJ (2013). Extracellular vesicles: biology and emerging therapeutic opportunities. Nat Rev Drug Discov.

[30] Zhang Y, Liu Y, Liu H, Tang WH (2019). Exosomes: biogenesis, biologic function and clinical potential. Cell Biosci.

[31] Henne WM, Buchkovich NJ, Emr SD (2011). The ESCRT pathway. Dev Cell.

[32] Eldh M, Ekstrom K, Valadi H, Sjostrand M, Olsson B, Jernas M (2010). Exosomes communicate protective messages during oxidative stress; possible role of exosomal shuttle RNA. PLoS One.

[33] Kim KM, Abdelmohsen K, Mustapic M, Kapogiannis D, Gorospe M (2017). RNA in extracellular vesicles. Wiley Interdiscip Rev RNA.

[34] Bang C, Thum T (2012). Exosomes: new players in cell-cell communication. Int J Biochem Cell Biol.

[35] Berchem G, Noman MZ, Bosseler M, Paggetti J, Baconnais S, Le Cam E (2016). Hypoxic tumor-derived microvesicles negatively regulate NK cell function by a mechanism involving TGF-beta and miR23a transfer. Oncoimmunology..

[36] Chen X, Ying X, Wang X, Wu X, Zhu Q, Wang X (2017). Exosomes derived from hypoxic epithelial ovarian cancer deliver microRNA-940 to induce macrophage M2 polarization. Oncol Rep.

[37] Tadokoro H, Umezu T, Ohyashiki K, Hirano T, Ohyashiki JH (2013). Exosomes derived from hypoxic leukemia cells enhance tube formation in endothelial cells. J Biol Chem.

[38] Yin Y, Cai X, Chen X, Liang H, Zhang Y, Li J (2014). Tumor-secreted miR-214 induces regulatory T cells: a major link between immune evasion and tumor growth. Cell Res.

[39] Yang M, Chen J, Su F, Yu B, Su F, Lin L (2011). Microvesicles secreted by macrophages shuttle invasion-potentiating microRNAs into breast cancer cells. Mol Cancer.

[40] Au Yeung CL, Co NN, Tsuruga T, Yeung TL, Kwan SY, Leung CS (2016). Exosomal transfer of stroma-derived miR21 confers paclitaxel resistance in ovarian cancer cells through targeting APAF1. Nat Commun.

[41] Lasser C, Alikhani VS, Ekstrom K, Eldh M, Paredes PT, Bossios A (2011). Human saliva, plasma and breast milk exosomes contain RNA: uptake by macrophages. J Transl Med.

[42] El-Andaloussi S, Lee Y, Lakhal-Littleton S, Li J, Seow Y, Gardiner C (2012). Exosome-mediated delivery of siRNA in vitro and in vivo. Nat Protoc.

[43] Haney MJ, Klyachko NL, Zhao Y, Gupta R, Plotnikova EG, He Z (2015). Exosomes as drug delivery vehicles for Parkinson’s disease therapy. J Control Release.

[44] Kim SM, Yang Y, Oh SJ, Hong Y, Seo M, Jang M (2017). Cancer-derived exosomes as a delivery platform of CRISPR/Cas9 confer cancer cell tropism-dependent targeting. J Control Release.

[45] Saari H, Lazaro-Ibanez E, Viitala T, Vuorimaa-Laukkanen E, Siljander P, Yliperttula M (2015). Microvesicle- and exosome-mediated drug delivery enhances the cytotoxicity of Paclitaxel in autologous prostate cancer cells. J Control Release.

[46] Crick F (1970). Central dogma of molecular biology. Nature..

[47] Dahlberg AE (1989). The functional role of ribosomal RNA in protein synthesis. Cell..

[48] Wilusz JE, Sunwoo H, Spector DL (2009). Long noncoding RNAs: functional surprises from the RNA world. Genes Dev.

[49] Holley RW, Apgar J, Everett GA, Madison JT, Marquisee M, Merrill SH (1965). Structure of a ribonucleic acid. Science..

[50] Wei JW, Huang K, Yang C, Kang CS (2017). Non-coding RNAs as regulators in epigenetics (Review). Oncol Rep.

[51] Pigati L, Yaddanapudi SC, Iyengar R, Kim DJ, Hearn SA, Danforth D (2010). Selective release of microRNA species from normal and malignant mammary epithelial cells. PLoS One.

[52] van Balkom BW, Eisele AS, Pegtel DM, Bervoets S, Verhaar MC (2015). Quantitative and qualitative analysis of small RNAs in human endothelial cells and exosomes provides insights into localized RNA processing, degradation and sorting. J Extracell Vesicles.

[53] Hannafon BN, Trigoso YD, Calloway CL, Zhao YD, Lum DH, Welm AL (2016). Plasma exosome microRNAs are indicative of breast cancer. Breast Cancer Res.

[54] Ragusa M, Statello L, Maugeri M, Barbagallo C, Passanisi R, Alhamdani MS (2014). Highly skewed distribution of miRNAs and proteins between colorectal cancer cells and their exosomes following cetuximab treatment: biomolecular, genetic and translational implications. Oncoscience..

[55] Kosaka N, Iguchi H, Hagiwara K, Yoshioka Y, Takeshita F, Ochiya T (2013). Neutral sphingomyelinase 2 (nSMase2)-dependent exosomal transfer of angiogenic microRNAs regulate cancer cell metastasis. J Biol Chem.

[56] Iavello A, Frech VS, Gai C, Deregibus MC, Quesenberry PJ, Camussi G (2016). Role of Alix in miRNA packaging during extracellular vesicle biogenesis. Int J Mol Med.

[57] Wu C, So J, Davis-Dusenbery BN, Qi HH, Bloch DB, Shi Y (2011). Hypoxia potentiates microRNA-mediated gene silencing through posttranslational modification of Argonaute2. Mol Cell Biol.

[58] Villarroya-Beltri C, Gutierrez-Vazquez C, Sanchez-Cabo F, Perez-Hernandez D, Vazquez J, Martin-Cofreces N (2013). Sumoylated hnRNPA2B1 controls the sorting of miRNAs into exosomes through binding to specific motifs. Nat Commun.

[59] Qu L, Ding J, Chen C, Wu ZJ, Liu B, Gao Y (2016). Exosome-transmitted lncARSR promotes sunitinib resistance in renal cancer by acting as a competing endogenous RNA. Cancer Cell.

[60] Kossinova OA, Gopanenko AV, Tamkovich SN, Krasheninina OA, Tupikin AE, Kiseleva E (2017). Cytosolic YB-1 and NSUN2 are the only proteins recognizing specific motifs present in mRNAs enriched in exosomes. Biochim Biophys Acta, Proteins Proteomics.

[61] Rauen T, Frye BC, Wang J, Raffetseder U, Alidousty C, En-Nia A (2016). Cold shock protein YB-1 is involved in hypoxia-dependent gene transcription. Biochem Biophys Res Commun.

[62] El-Naggar AM, Veinotte CJ, Cheng H, Grunewald TG, Negri GL, Somasekharan SP (2015). Translational activation of HIF1alpha by YB-1 promotes sarcoma metastasis. Cancer Cell.

[63] Li Y, Zheng Q, Bao C, Li S, Guo W, Zhao J (2015). Circular RNA is enriched and stable in exosomes: a promising biomarker for cancer diagnosis. Cell Res.

[64] Ostrowski M, Carmo NB, Krumeich S, Fanget I, Raposo G, Savina A (2010). Rab27a and Rab27b control different steps of the exosome secretion pathway. Nat Cell Biol.

[65] Dada LA, Novoa E, Lecuona E, Sun H, Sznajder JI (2007). Role of the small GTPase RhoA in the hypoxia-induced decrease of plasma membrane Na,K-ATPase in A549 cells. J Cell Sci.

[66] Dorayappan KDP, Wanner R, Wallbillich JJ, Saini U, Zingarelli R, Suarez AA (2018). Hypoxia-induced exosomes contribute to a more aggressive and chemoresistant ovarian cancer phenotype: a novel mechanism linking STAT3/Rab proteins. Oncogene..

[67] King HW, Michael MZ, Gleadle JM (2012). Hypoxic enhancement of exosome release by breast cancer cells. BMC Cancer.

[68] Booth AM, Fang Y, Fallon JK, Yang JM, Hildreth JE, Gould SJ (2006). Exosomes and HIV Gag bud from endosome-like domains of the T cell plasma membrane. J Cell Biol.

[69] Panigrahi GK, Praharaj PP, Peak TC, Long J, Singh R, Rhim JS (2018). Hypoxia-induced exosome secretion promotes survival of African-American and Caucasian prostate cancer cells. Sci Rep.

[70] Kucharzewska P, Christianson HC, Welch JE, Svensson KJ, Fredlund E, Ringner M (2013). Exosomes reflect the hypoxic status of glioma cells and mediate hypoxia-dependent activation of vascular cells during tumor development. Proc Natl Acad Sci U S A.

[71] Hsu YL, Hung JY, Chang WA, Lin YS, Pan YC, Tsai PH (2017). Hypoxic lung cancer-secreted exosomal miR-23a increased angiogenesis and vascular permeability by targeting prolyl hydroxylase and tight junction protein ZO-1. Oncogene..

[72] Li L, Li C, Wang S, Wang Z, Jiang J, Wang W (2016). Exosomes derived from hypoxic oral squamous cell carcinoma cells deliver miR-21 to normoxic cells to elicit a prometastatic phenotype. Cancer Res.

[73] Xue M, Chen W, Xiang A, Wang R, Chen H, Pan J (2017). Hypoxic exosomes facilitate bladder tumor growth and development through transferring long non-coding RNA-UCA1. Mol Cancer.

[74] Boeckel JN, Jae N, Heumuller AW, Chen W, Boon RA, Stellos K (2015). Identification and characterization of hypoxia-regulated endothelial circular RNA. Circ Res.

[75] Su H, Zou D, Sun Y, Dai Y (2019). Hypoxia-associated circDENND2A promotes glioma aggressiveness by sponging miR-625-5p. Cell Mol Biol Lett.

[76] Wang Y, Zhao R, Liu W, Wang Z, Rong J, Long X (2019). Exosomal circHIPK3 released from hypoxia-pretreated cardiomyocytes regulates oxidative damage in cardiac microvascular endothelial cells via the miR-29a/IGF-1 pathway. Oxidative Med Cell Longev.

[77] Lee S, Kopp F, Chang TC, Sataluri A, Chen B, Sivakumar S (2016). Noncoding RNA NORAD regulates genomic stability by sequestering PUMILIO proteins. Cell..

[78] Li LJ, Leng RX, Fan YG, Pan HF, Ye DQ (2017). Translation of noncoding RNAs: focus on lncRNAs, pri-miRNAs, and circRNAs. Exp Cell Res.

[79] Tripathi V, Ellis JD, Shen Z, Song DY, Pan Q, Watt AT (2010). The nuclear-retained noncoding RNA MALAT1 regulates alternative splicing by modulating SR splicing factor phosphorylation. Mol Cell.

[80] Wahlestedt C (2013). Targeting long non-coding RNA to therapeutically upregulate gene expression. Nat Rev Drug Discov.

[81] Guo X, Qiu W, Liu Q, Qian M, Wang S, Zhang Z, et al. Immunosuppressive effects of hypoxia-induced glioma exosomes through myeloid-derived suppressor cells via the miR-10a/Rora and miR-21/Pten pathways. Oncogene. 2018;37. 10.1038/s41388-018-0261-9 29713056

[82] Li L, Cao B, Liang X, Lu S, Luo H, Wang Z (2019). Microenvironmental oxygen pressure orchestrates an anti- and pro-tumoral gammadelta T cell equilibrium via tumor-derived exosomes. Oncogene..

[83] Dong C, Liu X, Wang H, Li J, Dai L, Li J (2019). Hypoxic non-small-cell lung cancer cell-derived exosomal miR-21 promotes resistance of normoxic cell to cisplatin. Onco Targets Ther.

[84] Chen X, Zhou J, Li X, Wang X, Lin Y, Wang X (2018). Exosomes derived from hypoxic epithelial ovarian cancer cells deliver microRNAs to macrophages and elicit a tumor-promoted phenotype. Cancer Lett.

[85] Tang T, Yang Z, Zhu Q, Wu Y, Sun K, Alahdal M, et al. Up-regulation of miR-210 induced by a hypoxic microenvironment promotes breast cancer stem cells metastasis, proliferation, and self-renewal by targeting E-cadherin. FASEB J. 2018:fj201801013R. 10.1096/fj.201801013R 30188754

[86] Zhang X, Sai B, Wang F, Wang L, Wang Y, Zheng L (2019). Hypoxic BMSC-derived exosomal miRNAs promote metastasis of lung cancer cells via STAT3-induced EMT. Mol Cancer.

[87] Sruthi TV, Edatt L, Raji GR, Kunhiraman H, Shankar SS, Shankar V (2018). Horizontal transfer of miR-23a from hypoxic tumor cell colonies can induce angiogenesis. J Cell Physiol.

[88] Li Z, He F, Yang Z, Cao X, Dai S, Zou J (2019). Exosomal miR-25-3p derived from hypoxia tumor mediates IL-6 secretion and stimulates cell viability and migration in breast cancer. RSC Adv.

[89] Yu Y, Min Z, Zhou Z, Linhong M, Tao R, Yan L, et al. Hypoxia-induced exosomes promote hepatocellular carcinoma proliferation and metastasis via miR-1273f transfer. Exp Cell Res. 2019;111649. 10.1016/j.yexcr.2019.111649 31562861

[90] Takahashi K, Yan IK, Haga H, Patel T. Modulation of hypoxia-signaling pathways by extracellular linc-RoR. J Cell Sci. 2014;127. 10.1242/jcs.141069 PMC3970562 24463816

[91] Takahashi K, Yan IK, Kogure T, Haga H, Patel T (2014). Extracellular vesicle-mediated transfer of long non-coding RNA ROR modulates chemosensitivity in human hepatocellular cancer. FEBS Open Bio.

[92] Mao G, Liu Y, Fang X, Liu Y, Fang L, Lin L (2015). Tumor-derived microRNA-494 promotes angiogenesis in non-small cell lung cancer. Angiogenesis..

[93] Park JE, Dutta B, Tse SW, Gupta N, Tan CF, Low JK (2019). Hypoxia-induced tumor exosomes promote M2-like macrophage polarization of infiltrating myeloid cells and microRNA-mediated metabolic shift. Oncogene..

[94] Umezu T, Tadokoro H, Azuma K, Yoshizawa S, Ohyashiki K, Ohyashiki JH (2014). Exosomal miR-135b shed from hypoxic multiple myeloma cells enhances angiogenesis by targeting factor-inhibiting HIF-1. Blood..

[95] Ye SB, Zhang H, Cai TT, Liu YN, Ni JJ, He J, et al. Exosomal miR-24-3p impedes T-cell function by targeting FGF11 and serves as a potential prognostic biomarker for nasopharyngeal carcinoma. J Pathol. 2016;240. 10.1002/path.4781 27538493

[96] Zhu X, Shen H, Yin X, Yang M, Wei H, Chen Q (2019). Macrophages derived exosomes deliver miR-223 to epithelial ovarian cancer cells to elicit a chemoresistant phenotype. J Exp Clin Cancer Res.

[97] Wang X, Luo G, Zhang K, Cao J, Huang C, Jiang T, et al. Hypoxic tumor-derived exosomal miR-301a mediates M2 macrophage polarization via PTEN/PI3Kgamma to promote pancreatic cancer metastasis. Cancer Res. 2018;78. 10.1158/0008-5472.CAN-17-3841 29880482

[98] Al Tameemi W, Dale TP, Al-Jumaily RMK, Forsyth NR (2019). Hypoxia-modified cancer cell metabolism. Front Cell Dev Biol.

[99] Bavelloni A, Ramazzotti G, Poli A, Piazzi M, Focaccia E, Blalock W (2017). MiRNA-210: a current overview. Anticancer Res.

[100] Wozniak M, Peczek L, Czernek L, Duchler M (2017). Analysis of the miRNA profiles of melanoma exosomes derived under normoxic and hypoxic culture conditions. Anticancer Res.

[101] Mittal V (2018). Epithelial mesenchymal transition in tumor metastasis. Annu Rev Pathol.

[102] Doktorova H, Hrabeta J, Khalil MA, Eckschlager T (2015). Hypoxia-induced chemoresistance in cancer cells: the role of not only HIF-1. Biomed Papers Med Facult Univ Palacky, Olomouc, Czechoslovakia.

[103] Abraham J, Salama NN, Azab AK (2015). The role of P-glycoprotein in drug resistance in multiple myeloma. Leuk Lymphoma.

[104] Fang G, Liu J, Wang Q, Huang X, Yang R, Pang Y (2017). MicroRNA-223-3p regulates ovarian cancer cell proliferation and invasion by targeting SOX11 expression. Int J Mol Sci.

[105] Laios A, O'Toole S, Flavin R, Martin C, Kelly L, Ring M (2008). Potential role of miR-9 and miR-223 in recurrent ovarian cancer. Mol Cancer.

[106] Gonzalez H, Hagerling C, Werb Z (2018). Roles of the immune system in cancer: from tumor initiation to metastatic progression. Genes Dev.

[107] Rao Q, Zuo B, Lu Z, Gao X, You A, Wu C (2016). Tumor-derived exosomes elicit tumor suppression in murine hepatocellular carcinoma models and humans in vitro. Hepatology..

[108] Ye SB, Li ZL, Luo DH, Huang BJ, Chen YS, Zhang XS (2014). Tumor-derived exosomes promote tumor progression and T-cell dysfunction through the regulation of enriched exosomal microRNAs in human nasopharyngeal carcinoma. Oncotarget..

[109] Day CL, Kaufmann DE, Kiepiela P, Brown JA, Moodley ES, Reddy S (2006). PD-1 expression on HIV-specific T cells is associated with T-cell exhaustion and disease progression. Nature..

[110] Ostrand-Rosenberg S (2018). Myeloid derived-suppressor cells: their role in cancer and obesity. Curr Opin Immunol.

[111] Tesi RJ (2019). MDSC; the most important cell you have never heard of. Trends Pharmacol Sci.

[112] Suetsuna F, Harata S, Yoshimura N (1991). Influence of the ultrasonic surgical aspirator on the dura and spinal cord. An electrohistologic study. Spine (Phila Pa 1976).

[113] Messmer MN, Netherby CS, Banik D, Abrams SI (2015). Tumor-induced myeloid dysfunction and its implications for cancer immunotherapy. Cancer Immunol Immunother.

[114] Mills CD, Kincaid K, Alt JM, Heilman MJ, Hill AM (2000). M-1/M-2 macrophages and the Th1/Th2 paradigm. J Immunol.

[115] Allavena P, Sica A, Garlanda C, Mantovani A (2008). The Yin-Yang of tumor-associated macrophages in neoplastic progression and immune surveillance. Immunol Rev.

[116] Viallard C, Larrivee B (2017). Tumor angiogenesis and vascular normalization: alternative therapeutic targets. Angiogenesis..

[117] Carmeliet P, Dor Y, Herbert JM, Fukumura D, Brusselmans K, Dewerchin M (1998). Role of HIF-1alpha in hypoxia-mediated apoptosis, cell proliferation and tumour angiogenesis. Nature..

[118] Babayan A, Pantel K (2018). Advances in liquid biopsy approaches for early detection and monitoring of cancer. Genome Med.

[119] Palmirotta R, Lovero D, Cafforio P, Felici C, Mannavola F, Pelle E (2018). Liquid biopsy of cancer: a multimodal diagnostic tool in clinical oncology. Ther Adv Med Oncol.

[120] Bardelli A, Pantel K (2017). Liquid biopsies, what we do not know (yet). Cancer Cell.

[121] Osti D, Del Bene M, Rappa G, Santos M, Matafora V, Richichi C (2019). Clinical significance of extracellular vesicles in plasma from glioblastoma patients. Clin Cancer Res.

[122] Vasconcelos MH, Caires HR, Abols A, Xavier CPR, Line A (2019). Extracellular vesicles as a novel source of biomarkers in liquid biopsies for monitoring cancer progression and drug resistance. Drug Resist Updat.

[123] Bjornetro T, Redalen KR, Meltzer S, Thusyanthan NS, Samiappan R, Jegerschold C (2019). An experimental strategy unveiling exosomal microRNAs 486-5p, 181a-5p and 30d-5p from hypoxic tumour cells as circulating indicators of high-risk rectal cancer. J Extracell Vesicles.

[124] Wang J, Zhou Y, Lu J, Sun Y, Xiao H, Liu M (2014). Combined detection of serum exosomal miR-21 and HOTAIR as diagnostic and prognostic biomarkers for laryngeal squamous cell carcinoma. Med Oncol.

[125] Wang YH, Ji J, Wang BC, Chen H, Yang ZH, Wang K (2018). Tumor-derived exosomal long noncoding RNAs as promising diagnostic biomarkers for prostate cancer. Cell Physiol Biochem.

[126] Pullan JE, Confeld MI, Osborn JK, Kim J, Sarkar K, Mallik S (2019). Exosomes as drug carriers for cancer therapy. Mol Pharm.

[127] Kamerkar S, LeBleu VS, Sugimoto H, Yang S, Ruivo CF, Melo SA (2017). Exosomes facilitate therapeutic targeting of oncogenic KRAS in pancreatic cancer. Nature..

[128] Yu LL, Zhu J, Liu JX, Jiang F, Ni WK, Qu LS (2018). A comparison of traditional and novel methods for the separation of exosomes from human samples. Biomed Res Int.

[129] Cheng L, Sharples RA, Scicluna BJ, Hill AF. Exosomes provide a protective and enriched source of miRNA for biomarker profiling compared to intracellular and cell-free blood. J Extracell Vesicles. 2014;3. 10.3402/jev.v3.23743 PMC3968297 24683445

